# QTL-based dissection of three key quality attributes in maize using double haploid populations

**DOI:** 10.3389/fpls.2025.1599530

**Published:** 2025-05-16

**Authors:** Zitian He, Jianping Wang, Jialei Li, Jianwei Li, Lei Chen, Xiaolei Zhang

**Affiliations:** ^1^ Crop Stress Molecular Biology Laboratory, Heilongjiang Bayi Agricultural University, Daqin, Heilongjiang, China; ^2^ Quality and Safety Institute of Agricultural Products, Heilongjiang Academy of Agricultural Sciences, Harbin, Heilongjiang, China; ^3^ Key Laboratory of Quality and Safety of Cereals and Their Products, State Administration for Market Regulation, Harbin, Heilongjiang, China; ^4^ Food Processing Institute, Heilongjiang Academy of Agricultural Sciences, Harbin, Heilongjiang, China; ^5^ Heilongjiang Academy of Agricultural Sciences, Harbin, Heilongjiang, China

**Keywords:** maize, starch, oil, lysine, QTLs, genetic analysis

## Abstract

**Introduction:**

Maize is a crucial source of nutrition, and the quality traits such as starch content, oil content, and lysine content are essential for meeting the demands of modern agricultural development. Understanding the genetic basis of these quality traits significantly contributes to improving maize yield and optimizing end-use quality. While previous studies have explored the genetic basis of these traits, further investigation into the quantitative trait loci (QTL) responsible for variations in starch content, oil content, and lysine content still requires additional attention.

**Methods:**

Double haploid (DH) populations were developed via a nested association mapping (NAM) design. Phenotypic data for starch, oil, and lysine content were collected using near-infrared spectroscopy and analyzed via ANOVA. Genotyping employed a 3K SNP panel, and genetic maps were constructed using QTL IciMapping. QTL analysis integrated single linkage mapping (SLM) and NAM approaches, with candidate genes identified via maizeGDB annotation and transcriptome data.

**Results:**

The broad-sense heritability of the populations with a range of 63.98-80.72% indicated the majority of starch content, oil content and lysine content variations were largely controlled by genetic factors. The genetic maps were constructed and a total of 47 QTLs were identified. The phenotypic variation explained (PVE) of the three traits is in a range of 2.60-17.24% which suggested that the genetic component of starch content, oil content and lysine content was controlled by many small effect QTLs. Five genes encoding key enzymes in regulation of starch, oil and lysine synthesis and metabolism located within QTLs were proposed as candidate genes in this study.

**Discussion:**

The information presented herein will establish a foundation for the investigation of candidate genes that regulate quality traits in maize kernels. These QTLs will prove beneficial for marker-assisted selection and gene pyramiding in breeding programs aimed at developing high-quality maize varieties.

## Introduction

Maize (*Zea mays* L.) is one of the most significant crops globally, contributing to 43% of total cereal production worldwide (Žilić et al., 2022). Beyond yield improvement, enhancing kernel quality traits-starch, oil, and lysine has emerged as a priority to meet rising demands for nutrient-dense crops and sustainable biorefineries (Planta and Messing, 2017; Mishra et al., 2025). Starch, constituting 70%–80% of kernel dry weight, directly determines caloric output and industrial utility, while oil (4%–5%) and lysine (0.13%–0.30%) are pivotal for nutritional value, particularly in livestock and human diets (Moro et al., 1996; Comparot-Moss and Denyer, 2009; Ranum et al., 2014; Zhang et al., 2019). This composition varies across genotypes and environments, highlighting the genetic complexity underlying carbohydrate and lipid allocation in kernels. Despite decades of genetic research, the interplay between these traits and their genetic networks remains poorly resolved, limiting holistic breeding strategies.

To elucidate the genetic variation in starch, oil, and lysine biosynthesis and metabolism, a multitude of QTL studies have been conducted over the past few years employing various mapping methods and diverse populations. These investigations have successfully identified numerous QTLs associated with quality traits in maize kernels. For instance, six QTLs associated with starch content in maize kernel were identified within a RIL population. Following the application of the bin-map method to refine the QTL intervals, seven genes emerged as candidate genes. Three of these genes encode enzymes are involved in non-starch metabolism, while four genes may function as direct regulators of starch biosynthesis (Wang et al., 2015). Subsequently, a total of 50 QTLs were identified, including 18 novel QTLs, through the integration of single linkage mapping (SLM), joint linkage mapping (JLM), and GWAS within a multi-parent population comprising six recombinant inbred line (RIL) populations (Hu et al., 2021). Many QTLs have been shown to regulate seed oil accumulation in a randomly mated F_2:3_ population derived from the cross between IHO and ILO (Alrefai et al., 1995; Laurie et al., 2004). These studies have demonstrated that oil content was controlled by numerous genes with individually small effects and mainly additive gene action (Yang et al., 2010). An important high-oil QTL, designated as *qHO6*, has been successfully cloned from chromosome 6. The candidate gene identified encodes an acyl-CoA:diacylglycerol acyltransferase (DGAT1-2), which is responsible for catalyzing the final step in oil synthesis (Zheng et al., 2008). The major QTL *Pal9*, which accounts for 42% of the phenotypic variation in palmitic acid content, was identified on maize chromosome 9 within a bi-parental segregating population. The candidate gene *Zmfatb*, which encodes acyl-ACP thioesterase, is associated with this QTL (Li et al., 2011b). In order to enhance breeding strategies aimed at achieving a balanced amino acid composition in maize kernels, several QTLs associated with lysine content or quality protein maize (QPM)-related traits have been examined in previous studies, including *o2* modifiers on chromosomes 5, 7, and 9 (Holding et al., 2011; Wu and Messing, 2014). Recent multi-parent population studies reveal that natural allelic diversity beyond o2 (e.g., *o7* and *fl2*) significantly impacts lysine biosynthesis, as observed in wheat and rice grain quality research (Hung et al., 2017; Kumar et al., 2020).

However, maize exhibits considerable phenotypic and genetic diversity (Ansaf et al., 2024). The molecular diversity in maize is estimated to be two- to fivefold greater than that found in other domesticated grass crops (Buckler et al., 2001). This extensive genetic variation has led to the observation that QTLs are often specialized within distinct populations and parent lines. Consequently, it motivates us to conduct further investigations using relevant germplasm to enhance our understanding of the genetic basis underlying quality traits. In this study, we constructed three DH populations using a NAM design with four quality parents. The genetic maps were developed and conducted subsequent analyses to investigate the genetic basis and identify QTLs associated with starch content, oil content, and lysine content. Additionally, we proposed key genes involved in related biosynthetic pathways within the QTL regions as candidate genes, which may provide valuable insights into the genetic foundation of quality traits in maize kernels and facilitate marker-assisted breeding for high-quality maize.

## Materials and methods

### Plant materials and growing environment

Four maize inbred lines (X987F, AJ5001, AJ7001, and AJ9010) from Maize Yufeng Biotechnology LLC were collected to construct three DH populations with X987F as the common parent, forming a NAM population. The plants were cultivated using a randomized complete block design across three different locations in 2022: Beijing (BJ, 40°08′N, 116°10′E), Neimeng (NM, 40°31′N, 107°05′E), and Liaoning (LN, 40°82’N, 123°56’E). Each line was grown in single-row plots, 250 cm in length, with 11 plants per row and 60 cm spacing between rows, under natural field conditions. The 11 plants in each row were self-pollinated, and 300 kernels were bulk-sampled from five moderate-sized ears, with an equal number of kernels collected from each ear. These bulk kernels were then used for phenotyping.

### Collection and analysis of phenotypic data

Phenotypic data were collected on three phenotypic quality traits of maize, including starch content, oil content, and lysine content. Specifically, a near-infrared reflectance (NIR) spectrometer (DA 7250, Perten Instruments Inc., Sweden) was utilized to assess the quality traits in maize kernels. Each sample underwent scanning three times to obtain an average value. The phenotypic variation of the quality traits was analyzed using R software version 4.0.1 with the “AOV” function (ANOVA). The ANOVA model employed is expressed as y = μ + α_g_ + β_e_ + ϵ, where α_g_ is the effect of the g^th^ line, β_e_ is the effect of the e^th^ environment, and ϵ is the error. All of the effects were considered to be random. These variance components were used to calculate the broad-sense heritability as *h^2^ =* σ*_g_* ^2^ */*(σ*_g_* ^2^ *+* σ*_ϵ_* ^2^/*e*), where σ*_g_^2^* is the genetic variance, σ*_ϵ_^2^* is the residual error, and *e* is the number of environments. To eliminate the influence of environmental effects, the best linear unbiased predictor (BLUP) value for each line was calculated using a linear mixed model that considered both genotype and environment as random effects in the R function “LME4,” and the trait BLUP values were used for subsequent analyses.

### Genotyping and construction of genetic linkage map

The genotype of the three DH populations with their parents was obtained by utilizing the GenoBaits Maize 1K marker panel (Mol Breeding Biotechnology Co., Ltd., Shijiazhuang, China). A total of 4,589 SNP markers were identified based on genotyping using a target sequencing platform. The minor allele frequency (MAF) and missing rate were estimated for each population, with SNPs exhibiting MAF < 0.1 or a missing rate > 0.6 being filtered out (Purcell et al., 2007). Following quality control measures, the polymorphic SNPs between the two parental lines were utilized to construct genetic linkage maps through the functions est.rf and est.map from the R/qtl package, employing the Kosambi mapping method (Broman et al., 2003). A joint linkage map for the three DH populations was developed using the CMP module in QTL IciMapping software (version 4.2).

### QTL mapping for maize kernel quality traits

Using the genetic linkage maps derived from three DH populations, SLM was conducted utilizing composite interval mapping as previously described (Zeng, 1994; Hu et al., 2021), which was implemented in Windows QTL Cartographer 2.5, according to the methodology of Wang et al. (2010) for each DH population. Model 6 of the Zmapqtl procedure (Basten et al., 1997) in the Composite Interval Mapping module was employed to identify QTLs across the genome by surveying intervals of 1.0 cM between markers within a window size of 10 cM. Forward–backward stepwise regression with five controlling markers was used to account for background contributions from adjacent markers. To determine the threshold logarithm of odds (LOD) value for putative QTLs, 1,000 permutations were performed for each trait in each DH population, and the resulting LOD score threshold ranged from 2.76 to 3.27 (α = 0.05). For simplification purposes, a LOD score threshold of 3.0 was adopted globally. The confidence interval concerning the position of the QTL was estimated using the 1.5-LOD support interval method as articulated in Liu’s study (Liu et al., 2017). Additionally, we utilized the R function “LM” to calculate total PVE by significant individual QTLs.

The NAM module in QTL IciMapping (version 4.2) was used to conduct the nested association mapping. The mapping approach employed was ICIM-ADD (Li et al., 2011a), with a step size set at 1.0 cM. In the same way, 1,000 permutations were performed for each trait. The resulting LOD score threshold ranged from 4.84 to 5.36 (α = 0.05), and we adopted a LOD score of 5.0 as the global threshold.

### Identification of candidate genes

All genes located within the QTL support interval were extracted based on their physical position using the *Zea mays* L. B73 reference genome version 4.0. The functional annotation of genes and information on starch, oil, and lysine metabolism pathways were obtained from the maizeGDB database (http://www.maizegdb.org/). The expression levels of candidate genes in different tissues are derived from public transcriptome data (Yi et al., 2019).

## Results

### Phenotypic variation and correlation analysis among three traits

**Figure 1:**
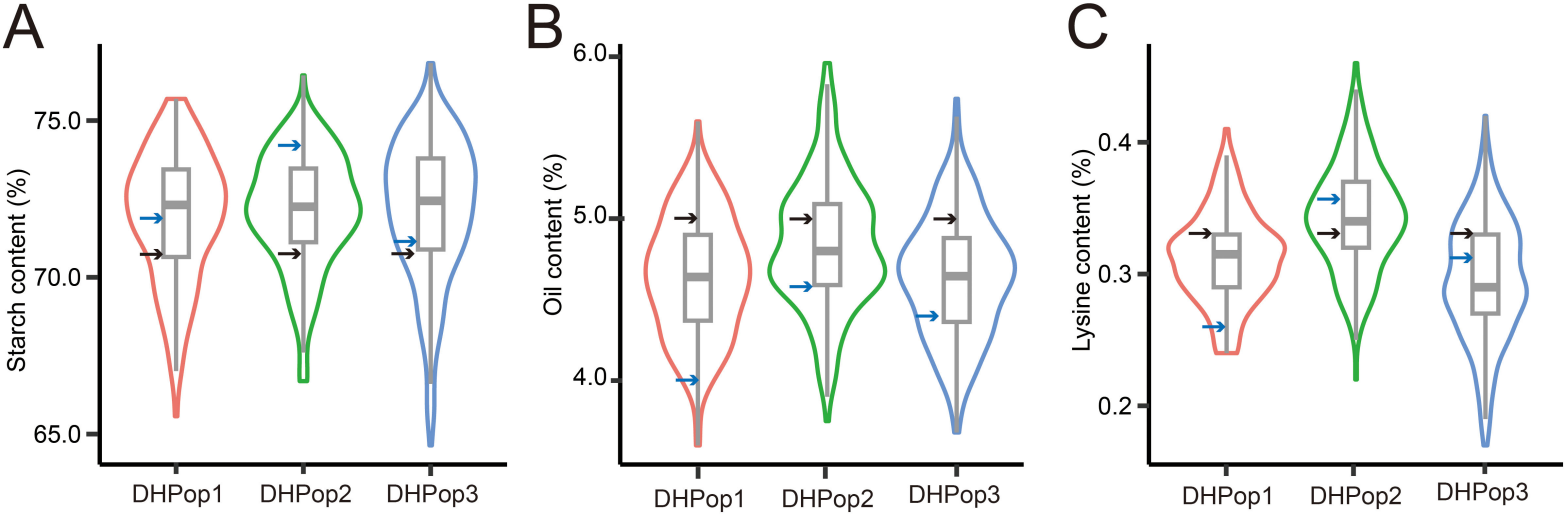
Phenotypic variation analysis of three traits. **(A–C)** Phenotypic variation in related to starch content, oil content, and lysine content among the three DH populations. The black arrows indicated the values for the common parent line X987F, while the blue arrows represented the values for the unique parent lines.

Four inbred lines exhibiting starch content ranging from 70.99% to 74.22%, oil content between 4.01% and 5.04%, and lysine content varying from 0.27% to 0.35% were collected for the development of three DH populations. The three DH populations, namely, DHPop1, DHPop2, and DHPop3, comprised 163, 219, and 320 lines, respectively (**Table 1**). The distributions of all traits within the DH populations showed continuous normality without significant skewness (**Figures 1A–C**). Analysis of variance (ANOVA) revealed that genotype variance exceeded environmental variance across almost all populations (**Table 1**), indicating that phenotypic variations were predominantly controlled by genetic factors. In addition, starch content, oil content, and lysine content exhibited average broad-sense heritabilities of 78.40%, 71.69%, and 74.89%, respectively (**Table 1**). Therefore, the abundant phenotypic variation along with high heritability observed in maize kernel quality traits across the three DH populations provides a solid genetic foundation for identifying new QTLs in maize.

**Table 1:** The phenotypic performance of four parents, variance and broad-sense heritability of starch content, oil content, and lysine content in the three DH populations.

Trait	Parameters	DH Populations					
		DHPop1		DHPop2		DHPop3	
Starch	Parents						
	Means ± SD (%)	X987F	70.99 ± 0.51				
		AJ5001	72.13 ± 0.47	AJ7001	74.22 ± 0.41	AJ9010	71.09 ± 0.89
	*p*-value ^a^	<0.001***		<0.0001****		<0.001***	
	DHs						
	Means ± SD (%)	71.93 ± 2.06		72.12 ± 1.82		72.16 ± 2.26	
	Range (%)	65.57–75.69		66.69–76.44		64.64–76.83	
	*h^2^* (%) ^e^	80.72%		75.63%		78.86%	
Oil	Parents						
	Means ± SD (%)	X987F	5.04 ± 0.13				
		AJ5001	4.01 ± 0.15	AJ7001	4.59 ± 0.17	AJ9010	4.44 ± 0.02
	*p*-value ^a^	<0.0001****		<0.001***		<0.001***	
	DHs						
	Means ± SD (%)	4.64 ± 0.37		4.84 ± 0.40		4.64 ± 0.37	
	Range (%)	3.60–5.60		3.75–5.96		3.68–5.74	
	*h^2^* (%) ^e^	78.93%		72.15%		63.98%	
Lysine	Parents						
	Means ± SD (%)	X987F	0.33 ± 0.03				
		AJ5001	0.27 ± 0.04	AJ7001	0.35 ± 0.02	AJ9010	0.31 ± 0.01
	*p*-value ^a^	<0.001***		<0.01**		<0.01**	
	DHs						
	Means ± SD (%)	0.31 ± 0.03		0.34 ± 0.04		0.30 ± 0.05	
	Range (%)	0.24–0.41		0.22–0.46		0.17–0.42	
	*h^2^* (%) ^e^	69.82%		77.9%		76.95%	

^a^ *p*-value based on a t-test evaluating two parental lines. ***p < 0.001, ****p < 0.0001; ^e^broad-sense heritability (*h^2^*).

### Genotyping and genetic linkage map

The three DH populations were genotyped by using the GenoBaits Maize 1K marker panel including 4,589 SNP markers. After quality control, DHPop1, DHPop2, and DHPop3 contained 1,266, 1,210, and 1,249 high-quality SNP markers, respectively, and covered all ten maize chromosomes. Based on the reference parental polymorphic loci, three linkage maps were independently constructed, with genetic map lengths of 728, 734, and 749 cM (**Supplementary Figure S1**). The average genetic distance between adjacent markers was 0.58, 0.61, and 0.62 cM in each DH population, respectively. The combined linkage map for the three DH populations using the CMP module in QTL IciMapping, included 1,993 SNP markers spanning a total genetic distance of 1,343 cM.

### Genetic architecture of quality traits dissected via two methods

**Figure 2:**
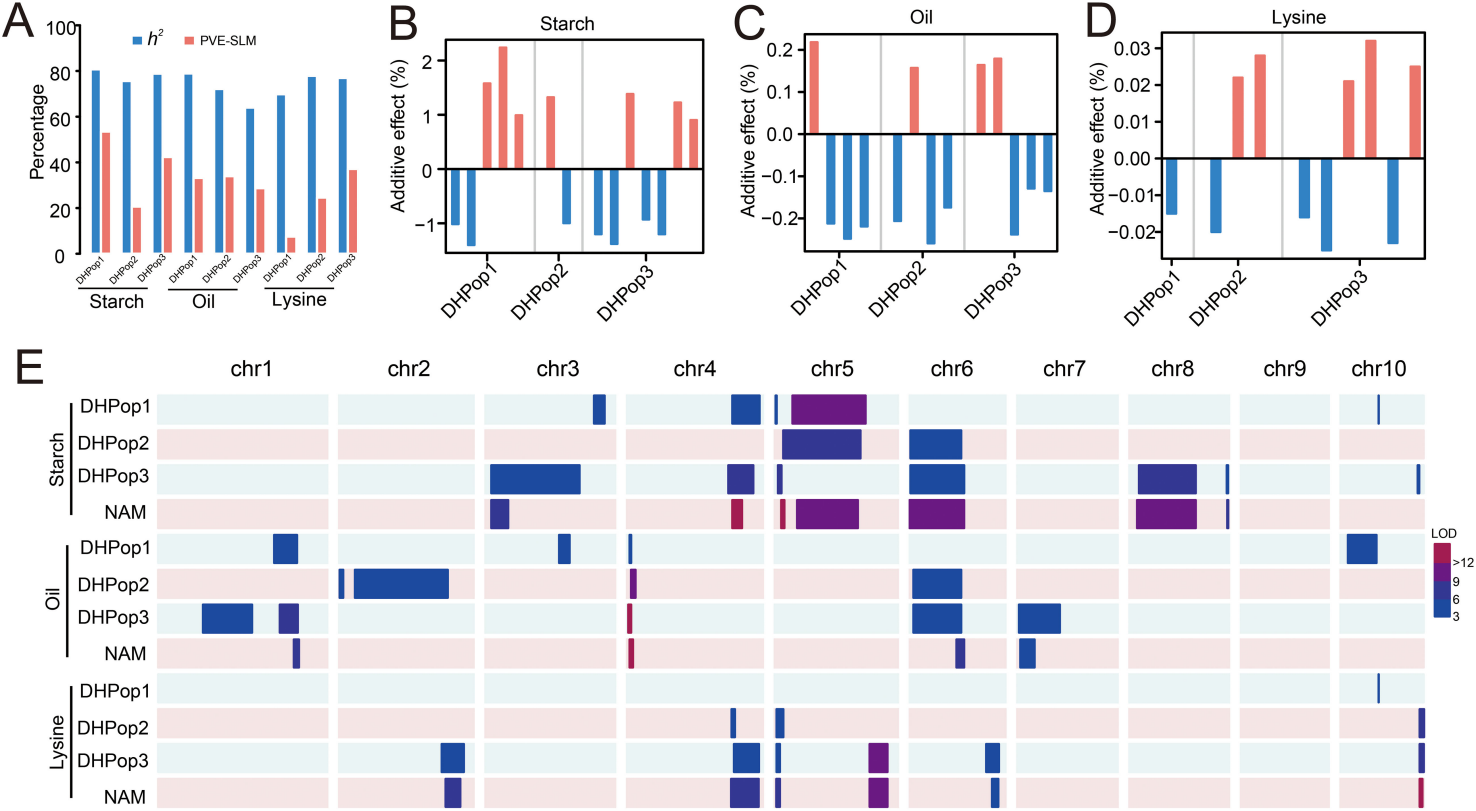
Summary of single QTLs for starch content, oil content, and lysine content identified by SLM and NAM analysis. **(A)** Broad-sense heritability (*h^2^*) and total PVE for single QTLs in each population. **(B–D)** Effect size and the origin of the increasing alleles of the identified single QTLs. Orange bars indicated that increasing alleles come from the unique parent lines, while blue bars indicated that increasing alleles come from the common parent. **(E)** Distribution of single QTLs on chromosomes.

We performed SLM analysis in each DH population with the composite interval mapping method (Zeng, 1994). In total, 37 QTLs for quality traits were detected, including 14 QTLs for starch content, 13 QTLs for oil content, and 10 QTLs for lysine content (**Supplementary Table S1**, **Supplementary Figure S2**). The 1.5-LOD supporting QTL interval averaged 46.26 cM, with a range of 1.7–168.2 cM. The total PVE by all identified QTLs in a population ranged from 20.67% to 53.51% for starch content, 28.71% to 33.98% for oil content, and 7.53% to 37.13% for lysine content (**Supplementary Table S1**). The PVE for the three traits showed far less than broad-sense heritability (**Figure 2A**), which suggested that some minor QTLs for these traits cannot be detected in bi-parent populations. Of these QTLs, 75.68% had the PVE <10% (**Supplementary Table S1**). For starch content, the PVE for each QTL ranged from 3.97% (*qSC14* in DHPop3) to 17.24% (*qSC04* in DHPop1), and only 28.57% (4/14) of the QTLs had large effects with PVE ≥ 10% (**Figure 2A**, **Supplementary Table S1**). For oil content, the PVE for each QTL ranged from 3.16% (*qOiL13* in DHPop3) to 14.27% (*qOiL07* in DHPop2), and only 23.08% (3/13) of the QTLs had large effects with PVE ≥ 10% (**Figure 2A**, **Supplementary Table S1**). For lysine content, the PVE for each QTL ranged from 5.14% (*qLys05* in DHPop3) to 12.06% (*qLys08* in DHPop3), and only 20.00% (2/10) of the QTLs had large effects with PVE ≥ 10% (**Figure 2A**, **Supplementary Table S1**). For each DH population, both parents in each DH population contained the alleles that increased starch content, oil content, and lysine content, respectively (**Figures 2B–D**, **Supplementary Table S1**). In addition, the QTL co-localization analysis among these three populations showed partial overlaps among more than two populations. Moreover, 10 QTLs uniquely detected in a given population underscored the genetic diversity of the founders of the DH populations (**Figure 2E**).

Subsequently, we identified a total of 17 QTLs using the NAM analysis, including seven QTLs for starch content, four QTLs for oil content, and six QTLs for lysine content (**Table 2**, **Supplementary Table S2**). The total PVE averaged 5.42%, with a range of 2.60%–9.72% in all populations for the three traits. Compared with the SLM results, the QTL interval was expectedly small, with 82.35% (14/17) of the QTL intervals falling within 50 Mb. The QTL co-localization analysis showed that almost all QTL from NAM were overlapping intervals with these from SLM, which indicates that the QTLs mined from the three DH populations established are representative (**Figure 2E**).

**Table 2:** Summary of QTLs for starch content, oil content, and lysine content identified via two methods.

	Starch content		Oil content		Lysine content	
Method	QTL number^a^	Total PVE (%)^b^	QTL number	Total PVE (%)	QTL number	Total PVE (%)
SLM	14 (2–7)	3.97–17.24	13 (4–5)	2.97–14.27	10 (1–6)	5.14–12.06
NAM	7	3.68–9.72	4	2.60–9.68	6	2.73–5.99

a The number of all QTLs identified via SLM in three DH populations is shown before brackets, while the range of QTLs identified in a given DH population are shown within the brackets;

b Phenotypic variance explained (PVE) by all single QTLs.

### Identification of candidate genes underlying the detected QTLs

**Figure 3:**
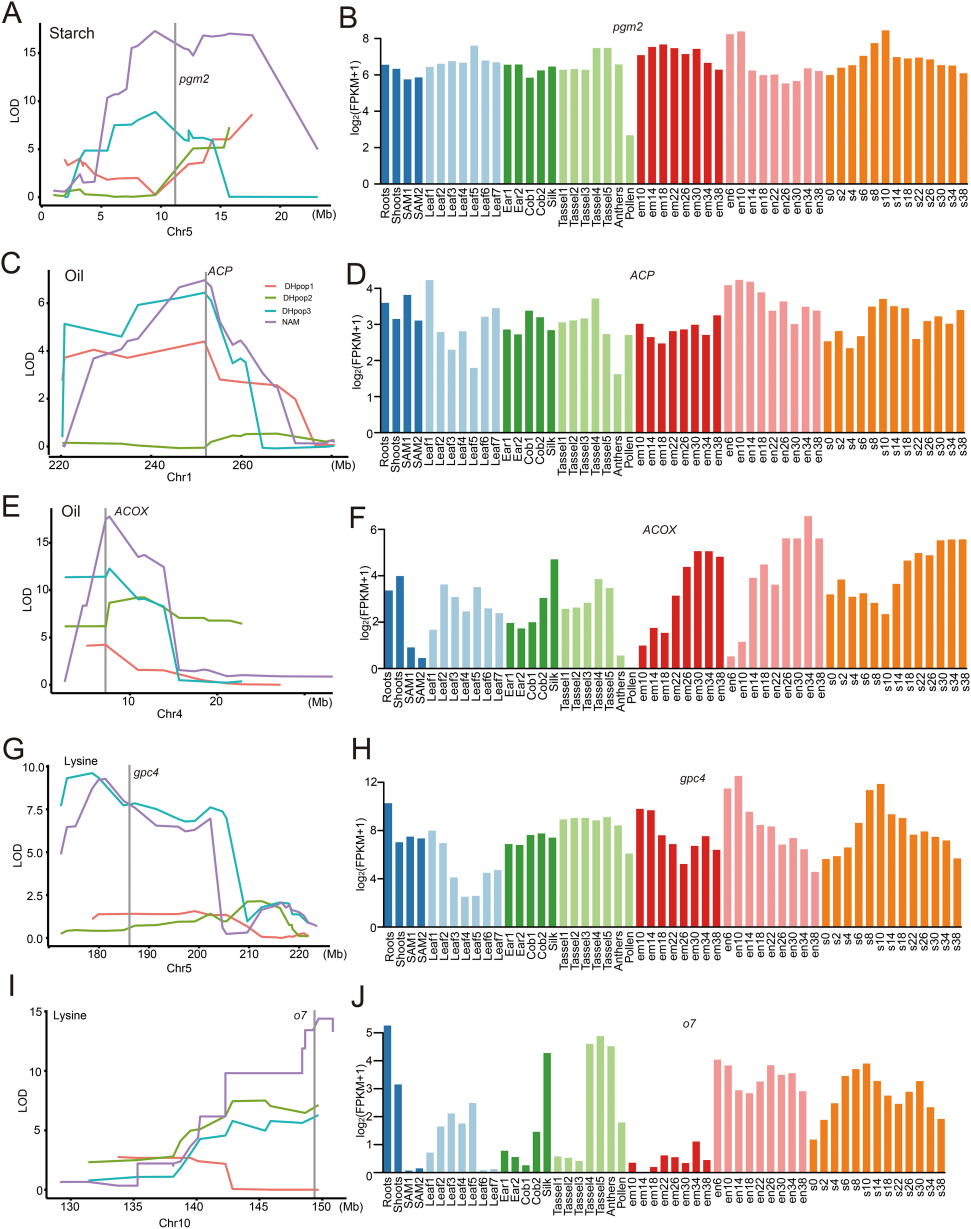
Co-localization of candidate genes **(A, C, E, G, I)** and their expression levels in different tissues from the public transcriptome data (Yi et al., 2019) **(B, D, F, H, J)**. SAM (shoot apical meristem) 1 and 2 : Vegetative Stages (V1 and V2); Leaf 1-7: the leaves of V1-V7; Ear 1-2: the ear of V1-V2; Cob 1-2: Reproductive Stages (R1-R2); Tassel 1-5: the tassel of V13-V18; em 10-38: the embryo of DAP (days after pollination) 10-38; en 6-38: the endosperm of DAP 6-38; s 0-38: the seed of DAP 6-38.

The genes located within the QTL support interval were extracted, resulting in the identification of 20,367 genes (**Supplementary Table S3**). According to the annotation in the MaizeGDB database (www.maizegdb.org), genes with the most relevant functional annotation information was nominated as the candidate genes. Phosphoglucomutase2 protein-coding gene *pgm2* was the most abundant annotations gene for starch content positioned in 11,195,010–11,201,017 interval of chromosome 5 (**Figure 3A**, **Supplementary Table S3**). Expression pattern analysis showed that *pgm2* was expressed in all tissues of maize, and the expression levels were relatively the same except in pollen (**Figure 3B**). There were two annotations genes for oil content. Acyl carrier protein coding gene *ACP* was positioned in 252129992–252138538 interval of chromosome 1 and expressed in all tissues (**Figures 3C, D**; **Supplementary Table S3**). Acyl-coenzyme A oxidase coding gene *ACOX* was positioned in 7209473–7214489 interval of chromosome 4 (**Figure 3E**, **Supplementary Table S3**). *ACOX* was also expressed in almost all tissues of maize except in pollen (**Figure 3F**). In addition, the expression levels in the embryo and endosperm vary with different developmental stages (**Figure 3F**). For lysine content, glyceraldehyde-3-phosphate dehydrogenase 4 coding gene *gpc4* and Acyl-activating enzyme-like protein coding gene *o7* were positioned in 186104976–186109690 interval of chromosome 5 and 149382212–149384169 interval of chromosome 10, respectively (**Figure 3G, I**; **Supplementary Table S3**). *gpc4* was expressed in all tissues of maize, and the expression level of *o7* was significantly lower in the shoot apical meristem (SAM), ear, cob, and embryo tissues compared to other tissues (**Figures 3H, J**).

## Discussion

### QTL mapping precision

SNP markers represent the most prevalent variations within genomes, and their application in plant breeding has significantly enhanced the precision of QTL mapping and genetic analysis (Bhattramakki et al., 2002; Mammadov et al., 2012; Kaur et al., 2021). By conditioning linked markers during testing, the sensitivity of the test statistic to the positions of individual QTLs is heightened, thus improving the accuracy of QTL mapping (Zeng, 1994). With advancements in sequencing technology, an increasing array of molecular markers has been utilized for QTL mapping, leading to substantial improvements in its accuracy (Schnable et al., 2009; Chia et al., 2012; Bukowski et al., 2018; Fang et al., 2021). In this study, the average physical intervals of the QTLs were 42.11 Mb; 29.79% (14/47) spanned physical intervals of <10 Mb, and 68.09% (32/47) spanned <50 Mb. These findings indicate a considerable enhancement in resolution due to both the large number of employed markers and the suitable population type used in this research. The resolution is likely on the order of 2–3 cM, since pairs of markers that are farther apart rarely exhibit significant levels of linkage disequilibrium.

### Genetic basis of maize kernel quality traits in the DH and NAM populations

The maize kernel quality traits including starch content, oil content, and lysine content are complex quantitative traits, and the genetic basis are still unresolved issues at present (Holding et al., 2008; Huang et al., 2021). A set of parameters that elucidates the genetic component underlying trait variation within or among populations will influence the genetic architecture of quantitative traits. Mapping populations represent one of the critical parameters capable of capturing genetic diversity and possessing the power to detect QTLs with small effects (Odell et al., 2022). DH populations have the advantages of retaining homozygosity and genetically identical replicates (Bordes et al., 2006; Foiada et al., 2015; Yan et al., 2017; Chaikam et al., 2019) and nested association mapping simultaneously exploits the advantages of both linkage and association mapping and has been successfully applied to investigate the genetic basis of complex quantitative traits in maize (Yu et al., 2008; Gage et al., 2020). In this study, we developed a NAM population formed by three DH populations for detection of QTLs and identification of candidate genes. The results of the phenotypic and genetic detection showed that there was a wide phenotypic variation extent in starch content (64.64%–76.83%), oil content (3.60-5.96%), and lysine content (0.17-0.46%) and high broad-sense heritability in the DH populations. It illustrated that these traits were mainly controlled by genetic factors. Totally, 47 QTLs were found in the three DH populations and distributed on chromosomes 1–10. Among the identified QTLs, three QTLs (*qSC02* in DHPop1 and *qSC09* in DHPop3 for starch content, *qLys06* in DHPop3 for lysine content) spanned a 104.93 Mb physical interval on chromosome 4. Four QTLs (*qSC07* in DHPop2 and *qSC11* in DHPop3 for starch content, *qOiL08* in DHPop3 and *qOiL12* in DHPop3 spanned 86.51 Mb physical interval on chromosome 6. These results indicated that there is cross-over in the regulatory network of starch content, oil content, and lysine content in maize kernel. The average PVE per QTL was 7.89% using SLM in each DH population, whereas it was 5.42% using NAM method across three DH populations. Similar to previous studies, our findings align with the quantitative nature of these quality traits, which are acknowledged to be governed by a multitude of genes and QTLs with minor effects.

### Candidate genes underlying quality traits QTLs

For starch content, *pgm2* (Zm00001d013428) located in *qSC10* was the most abundant annotations among the annotated genes (**Figure 3A**), which encoded phosphoglucomutase 2. Phosphoglucomutase (PGM) is a phosphoenzyme (EC 5.4.2.2) and catalyzes an important trafficking point in carbohydrate metabolism in cells of prokaryotic and eukaryotic organisms (Manjunath et al., 1998). In maize, cytosolic isozymes of PGM are encoded by *pgm1* and *pgm2* (Goodman et al., 1980; Stuber and Goodman, 1983), and the existing levels of PGM are sufficient to maintain the flux of Glc-1-phosphate into glycolysis under O_2_ deprivation (Manjunath et al., 1998).

There were three most abundant annotations genes for oil content (**Figure 3A**). *ACP* (Zm00001d033149) in *qOiL10* interval was annotated to be one of the important acyl carrier protein encoding gene. ACP is linked to the malonyl group via a transacylation reaction when acetyl-CoA is carboxylated to malonyl-CoA and participates in *de novo* fatty acid synthesis in plants and prokaryotes (Kalinger and Rowland, 2023). In *qOiL03* interval, there were two abundant annotations genes both related to lipid synthesis metabolic pathways. *ACOX* (Zm00001d048890) was identified as acyl-coenzyme oxidase encoding gene that involved in the fatty acid β-oxidation pathway in plant peroxisomes (Li et al., 2024; Ruan et al., 2024). ACOX family members may specifically recognize distinct chain lengths of fatty acids and catalyze the first step of peroxisomal fatty acid β-oxidation to converse fatty acyl-CoAs to trans-2-enoyl CoAs (Chu et al., 1994). This step is thought to be critical the rate of carbon flux in the β-oxidation pathway (Pinfield-Wells et al., 2005).

*Zm00001d017121* in *qLys08* is one of the small multi-gene family, which encoded a glyceraldehyde-3-phosphate dehydrogenase. In maize cytoplasm, glyceraldehyde-3-phosphate dehydrogenase protein is synthesized in roots during anoxic conditions and is known to be one of the “anaerobic polypeptides” (Manjunath and Sachs, 1997). In plants, glyceraldehyde-3-phosphate dehydrogenase (GAPDH), a key enzyme in the glycolytic pathway, reversibly converts the glyceraldehyde-3-phosphate to 1,3-bisphosphoglycerate by coupling with the reduction in NAD1 to NADH (Sirover, 1997) and plays an important role in several cellular processes, including plant hormone signaling, plant development, and transcriptional regulation (Kim et al., 2024). *Zm00001d026649* in *qLys04* was annotated as *opaque endosperm7* (*o7*) gene. *o7* is one of the three most important high-lysine mutants in maize, alongside *opaque2* (*o2*) and *floury2* (*fl2*) (Misra et al., 1972; Miclaus et al., 2011). The *o7* mutant endosperm was characterized as having significantly more lysine due to a general reduction in zein levels and an increase in other lysine-rich proteins like the albumins and globulins (Misra et al., 1972; Azevedo et al., 2004).

## Conclusion

In this study, four maize inbred lines were used in a NAM design to establish three DH populations. The kernel starch content, oil content, and lysine content exhibited continuously and approximately normal distribution. The analysis of broad-sense heritability indicated that the majority of the three quality traits variations were largely controlled by genetic factors. Nine major and 38 minor effect QTLs were identified based on the genetic linkage map with LOD threshold of 3.00 with PVE in the range of 2.60%–17.24%, which suggested that the genetic component of starch content, oil content, and lysine content was controlled by many small effect QTLs. Furthermore, five main genes that were involved in starch, oil, and lysine synthesis and metabolism pathways were the causal candidate genes underlying the identified QTLs. These QTLs in this study will facilitate the exploration of candidate genes that regulate the quality characteristics of maize kernels. The advancement will also benefit molecular breeding programs that utilize marker-assisted selection to develop maize varieties with optimal quality traits.

## Data Availability

The datasets presented in this study can be found in online repositories. The names of the repository/repositories and accession number(s) can be found in the article/**Supplementary Material**.
