# Supplementary material for: QTL-based dissection of three key quality attributes in maize using double haploid populations
Source: Front Plant Sci. 2025 May 16;16:1599530. doi: 10.3389/fpls.2025.1599530 (PMC12122741; doi:10.3389/fpls.2025.1599530)
Supplement: Supplementary file 2 [file Table2.docx]

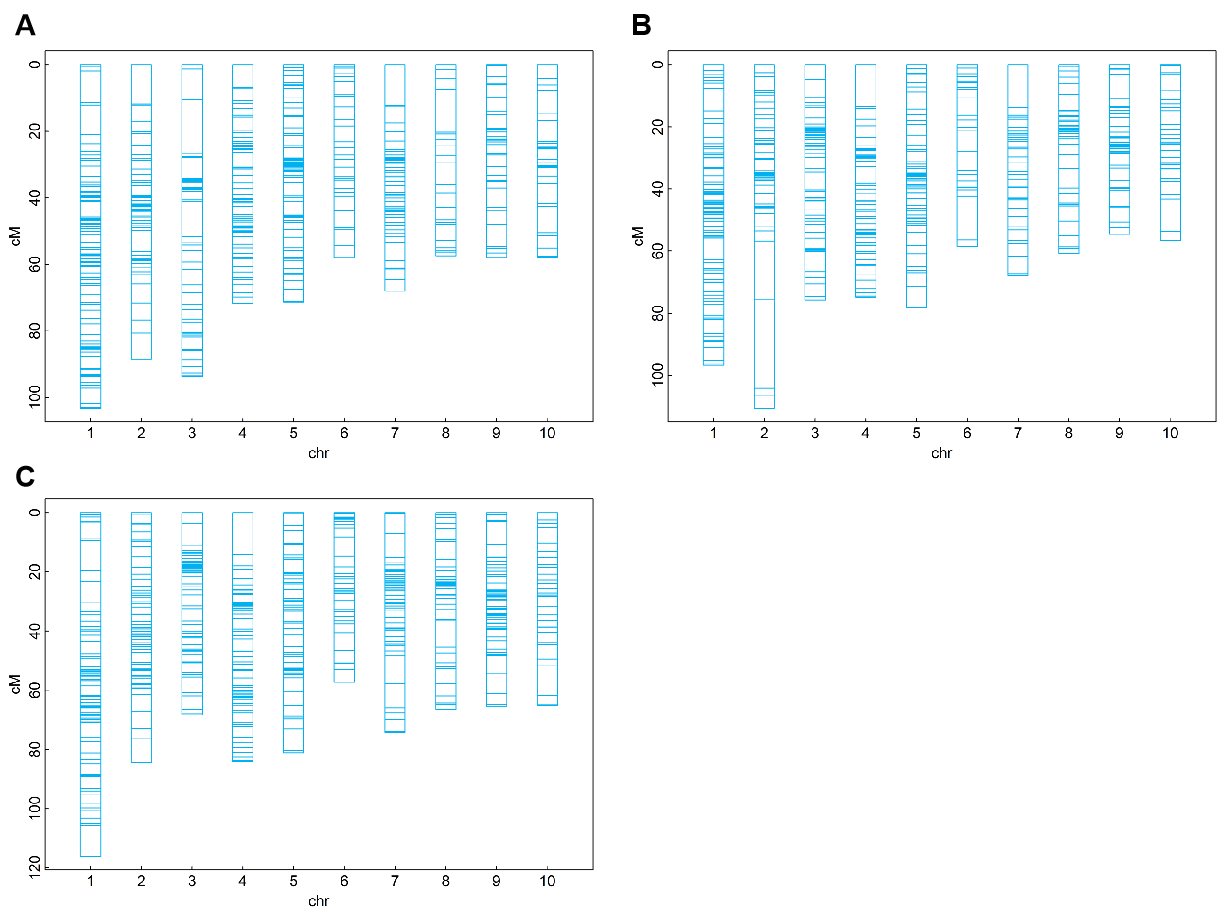


**Supplementary FIGURE S1.** Linkage maps of three DH populations. The horizontal light blue bars on each chromosome showed the genetic position of each SNP.  A-C designated DHPop1, DHPop2 and DHPop3, respectively.
