# Supplementary material for: QTL-based dissection of three key quality attributes in maize using double haploid populations
Source: Front Plant Sci. 2025 May 16;16:1599530. doi: 10.3389/fpls.2025.1599530 (PMC12122741; doi:10.3389/fpls.2025.1599530)
Supplement: Supplementary file 3 [file Table3.docx]

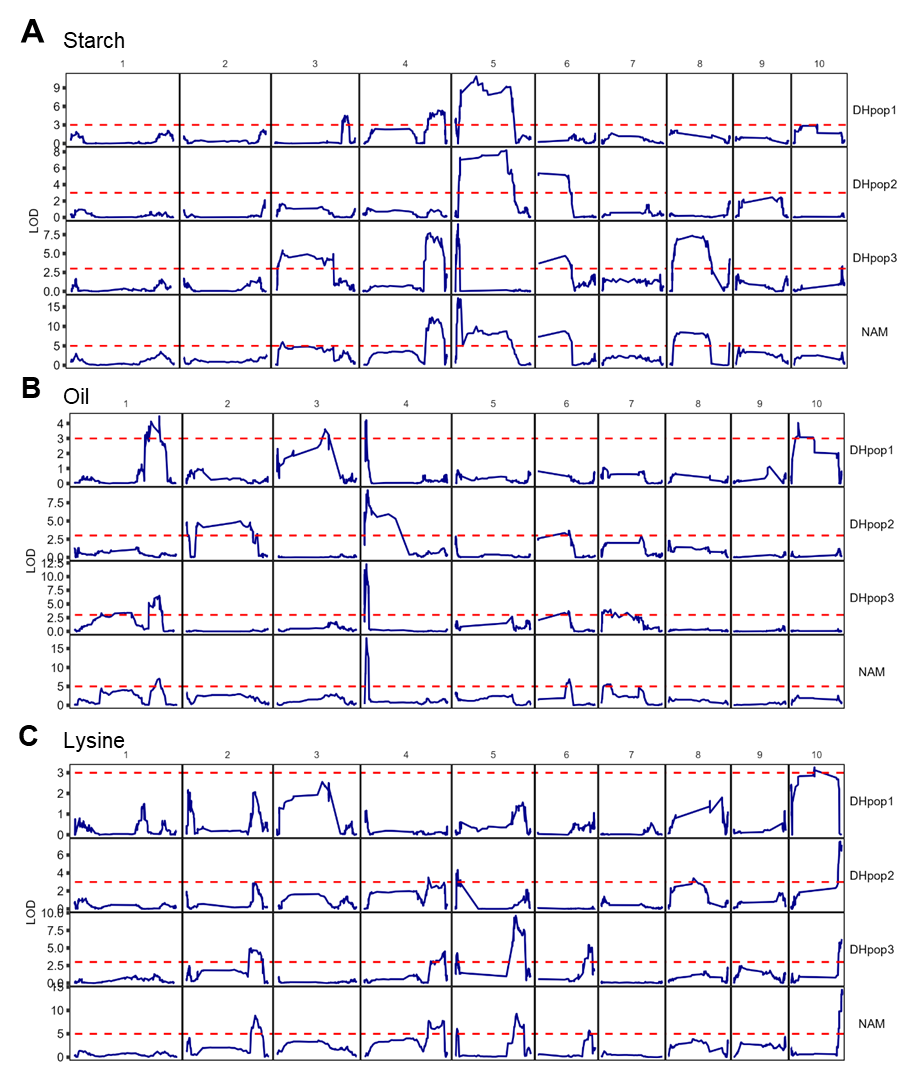


**Supplementary FIGURE S2.** The LOD profile of QTLs across the entire genome in the three DH populations and nested association mapping (NAM). Each picture displays a LOD score (y-axis) against the physical position (x-axis) of markers. A-C designated starch, oil and lysine, respectively.
